# Caffeic Acid-Grafted PLGA as a Novel Material for the Design of Fluvastatin-Eluting Nanoparticles for the Prevention of Neointimal Hyperplasia

**DOI:** 10.1021/acs.molpharmaceut.2c00693

**Published:** 2022-10-17

**Authors:** Stefano Bellosta, Francesca Selmin, Giulia Magri, Silvia Castiglioni, Patrizia Procacci, Patrizia Sartori, Edoardo Scarpa, Valerio Tolva, Clara Rossi, Francesco Puoci, Loris Rizzello, Francesco Cilurzo

**Affiliations:** † Dept. Pharmacological and Biomolecular Sciences, Università Degli Studi di Milan, Via G. Balzaretti 9, Milan20133, Italy; ‡ Dept of Pharmaceutical Sciences, Università Degli Studi di Milano, via G. Colombo, 71, Milan20133, Italy; § Dept of Biomedical Sciences for Health, Università Degli Studi di Milano, via G. Colombo, 71, Milan20133, Italy; ∥ National Institute of Molecular Genetics (INGM), via F. Sforza, 35, Milan20122, Italy; ⊥ Struttura Complessa di Chirurgia Vascolare, Fondazione “A. De Gasperis”, ASST Grande Ospedale Metropolitano Niguarda, Piazza Ospedale Maggiore 3, Milan20162, Italy; # Dept of Pharmacy, Health and Nutritional Sciences, University of Calabria, Rende87036, Cosenza, Italy

**Keywords:** confocal microscopy, DIL, prolonged release, silver nanoparticle, fluvastatin, resveratrol

## Abstract

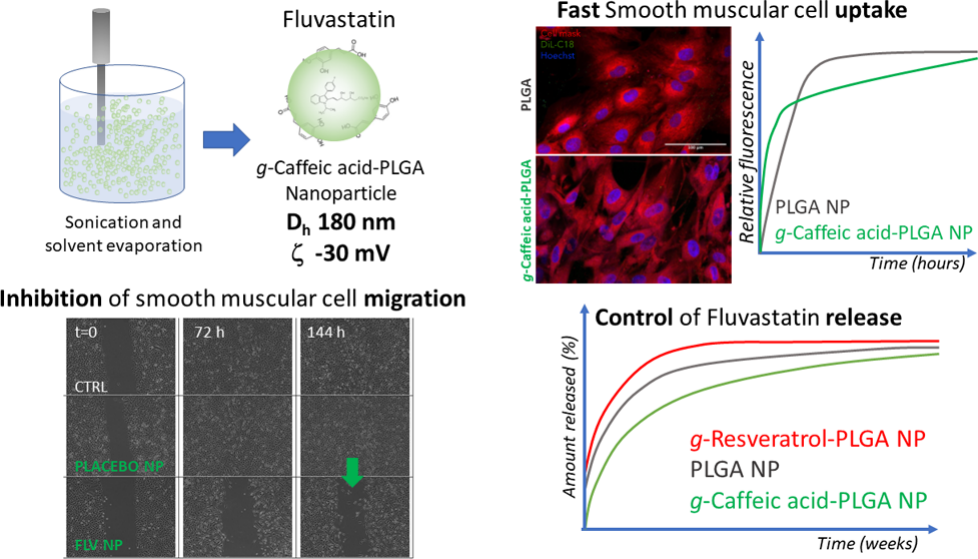

Drug-eluting nanoparticles (NPs) administered by an eluting balloon represent a novel tool to prevent restenosis after angioplasty, even if the selection of the suitable drug and biodegradable material is still a matter of debate. Herein, we provide the proof of concept of the use of a novel material obtained by combining the grafting of caffeic acid or resveratrol on a poly(lactide-*co*-glycolide) backbone (*g*-CA-PLGA or *g*-RV-PLGA) and the pleiotropic effects of fluvastatin chosen because of its low lipophilic profile which is challenging for the encapsulation in NPs and delivery to the artery wall cells. NPs made of such materials are biocompatible with macrophages, human smooth muscle cells (SMCs), and endothelial cells (ECs). Their cellular uptake is demonstrated and quantified by confocal microscopy using fluorescent NPs, while their distribution in the cytoplasm is verified by TEM images using NPs stained with an Ag–PVP probe appositely synthetized. *g*-CA-PLGA assures the best control of the FLV release from NP sizing around 180 nm and the faster SMC uptake, as demonstrated by confocal analyses. Interestingly and surprisingly, *g*-CA-PLGA improves the FLV efficacy to inhibit the SMC migration, without altering its effects on EC proliferation and migration. The improved trophism of NPs toward SMCs, combined with the excellent biocompatibility and low modification of the microenvironment pH upon polymer degradation, makes *g*-CA-PLGA a suitable material for the design of drug-eluting balloons.

## 1 Introduction

Cardiovascular diseases are still the primary causes of morbidity and mortality worldwide. Indeed, according to the WHO, the ischemic heart disease is responsible for 16% of the world’s total deaths. Since the year 2000, the largest increase in deaths has been, for this disease, rising by more than 2 million to 8.9 million deaths in 2019. Stroke is the second leading cause of death, being responsible for approximately 11% of total deaths.

Cardiovascular diseases are known to have a complex and multifaceted etiology. A lesioned endothelial layer drives the evolution of the “atheroma” by multiple pathways including the following: (1) chemotaxis and recruitment of circulating monocytes, (2) inhibition of macrophages migration through the arterial intima, (3) enhanced uptake of low-density lipoproteins by macrophages, *en route* to foam cell formation, and (4) proliferation of vascular smooth muscle cells (SMCs) with consequent intimal layer thickening. When the atherosclerotic lesion becomes “vulnerable” in terms of embolic or thrombotic events, a surgical procedure is mandatory to avoid clinical consequences.^1^ Even if endovascular procedures have become less invasive with a shorter recovery time, percutaneous transluminal angioplasty (PTA) suffers from a high rate of restenosis (about 40% of the cases within 6 months). Moreover, stent placement, that exhibits early- and mid-term good results data, shows long-term restenosis at rates of about 30 and 12–15% in the case of bare metal and drug-eluting stents, respectively.^2^

Upon PTA procedures, very complex cellular processes responsible for restenosis can occur, including local reendothelialization and vascular remodeling mediated by the infiltration of a variety of inflammatory cells, cytokines, and growth factors. The pathophysiology of post-PTA restenosis involves neointimal formation that develops through three phases: thrombosis (within 24 h), recruitment (3–8 days), and proliferation of SMC, which starts on day 8 after PTA.^3^ Poor reendothelialization and excessive migration and proliferation of vascular SMC in the tunica media peak at 6 months after stenting.^4^ Consequently, patients at high risk of restenosis are preferentially treated with plain balloons or with drug-eluting balloons (DEBs).^5,6^

In general, DEBs are coated with an anti-proliferative drug, mostly paclitaxel or sirolimus.^7^ The treatment strategy entails that the drug is rapidly and homogeneously transferred to the vessel wall during balloon inflation. However, drug lost in balloon transit or inflation may exceed 80% of the intended drug dose, and crystalline/non-crystallin amorphous coatings may be associated with particulate debris, distal microembolization, and downstream tissue ischemia and infarction.^8^ To solve these drawbacks and improve the efficacy of the pharmacological treatment, polymeric nanoparticles (NPs) have been proposed to be administered intramurally by DEB because they can localize and sustain the drug concentration at the injured vessel.^9−11^ One of the most accepted polymer families used to design implantable drug delivery systems is poly(lactide-*co*-glycolide) (PLGA), although there are several concerns on its use, including the formation of acidic degradation products and a moderate cytocompatibility, which in turn can lead to an intense inflammatory reaction^12^ and a neointimal thickening.^13−15^ A possible approach to improve PLGA biocompatibility is the grafting of an anti-oxidant on PLGA (*g*-AA-PLGA). Indeed, *g*-AA-PLGA enabled to reduce the variation of environmental pH upon polymer degradation,^16^ providing anti-oxidant properties and improving the encapsulation efficiency of hydrophilic drugs with respect to the naïve PLGA.^17^

The widely studied and used compounds to limit the risk of restenosis are anti-proliferative drugs, such as tacrolimus and sirolimus.^18−21^ However, in the last years, emerging evidence has been suggesting that other therapeutic agents can halt or at least slow down stenosis progression.^11^ As a matter of fact, statins have effects that go beyond their mechanism of action, the so-called *pleiotropic effects*.^22−25^ Besides lowering the serum cholesterol level, they possess anti-inflammatory, anti-thrombotic, anti-oxidant, and anti-mitotic effects, in addition to inhibition of SMC proliferation^24,26,27^ and matrix metalloproteinase secretion.^28−30^ Preprocedural statin therapy may reduce peri- and post-PTA myonecrosis and the need for repeated revascularization; meanwhile, statin-eluting stents inhibit restenosis in animal models.^3,31^

This work aims to explore the potentialities of fluvastatin sodium salt (FLV)-eluting NPs made of caffeic acid (*g*-CA-PLGA) or resveratrol (*g*-RV-PLGA) in the prevention of restenosis. FLV appears of interest because the high water solubility makes the encapsulation in PLGA NPs and delivery to the artery wall challenging.

First, the polymer biocompatibility was studied because this feature is a key functional requirement of next-generation medical devices. Then, NP uptake was studied in SMCs, endothelial cells, and macrophages, selected as a representative of the cell population in the blood vessel, by confocal microscopy and TEM. In this case, NPs with an electrodense core were also prepared by a probe made of a combination of Ag and PVP (Ag–PVP) appositely developed. Finally, the antiproliferative potency of FLV-eluting NPs was investigated in cellular models.

## 2 Experimental Section

### 2.1 Materials

Capped poly (d,l-lactide-*co*-glycolide) with a co-monomer ratio of 50:50, PURASORB PDLG 5002 (PLGA), was obtained from Corbion PURAC (NL). Caffeic acid (CA), resveratrol (RV), hydrogen peroxide (H_2_O_2_), ascorbic acid (AA), 2,2′-diphenyl-1-picrylhydrazyl radical (DPPH), poly(vinyl pyrrolidone) K30 (PVP), silver nitrate (AgNO_3_), and nitric acid (HNO_3_) were purchased from Sigma-Aldrich (I); fluvastatin sodium salt (FLV) was a gift from Novartis (I). DiIC18(5)-DS (1,1′-dioctadecyl-3,3,3′,3′-tetramethylindodicarbocyanine-5,5′-disulfonic acid) (DIL) was purchased from Thermo Fisher (I). All solvents were of analytical grade, unless specified.

### 2.2 Synthesis and Characterization of *g*-AA-PLGA

In a 100 mL round-bottom flask, 0.5 g of PLGA was dissolved in 5 mL of THF and the obtained solution was evaporated leading to the formation of a thin polymeric film. After the addition of 50 mL of a hydrogen peroxide (H_2_O_2_) solution (1.0 M) and 1.2 g of ascorbic acid, the reaction was allowed to stand for 30 min. Then, the H_2_O_2_/AA solution was removed and replaced with 50 mL of a mixture consisting of H_2_O_2_ (2.0 M) and ethanol (1:1 v/v) containing 1.2 g of ascorbic acid and 0.8 mmol of the anti-oxidant agent (CA or RV). The reaction mixture was maintained at 25 °C under atmospheric air, and, after 24 h, the obtained functionalized film was purified by washing with distilled water and ethanol and dried under vacuum for 24 h at room temperature.

Polymer molecular weights were determined by using a HP1100 ChemStation (Agilent, Santa Clara, CA, USA) equipped with a combination of two columns: μStyragel Toluene 104 Å 7.8 × 300 mm and μStyragel Toluene 103 Å 7.8 × 300 mm (Waters, Milan, I). Chromatographic conditions. Mobile phase: THF; flow rate: 1.0 mL·min^–1^; detector: refractive index signal; injection volume: 20 μL. The molecular weight (*M*_w_) and polydispersity index (PI) of each sample was calculated using a calibration curve made with monodisperse polystyrene standards, *M*_w_ ranging from 1000 to 45,000 Da. A differential scanning calorimeter equipped with a refrigerated cooling system (DSC 1 Stare System, METTLER TOLEDO, CH) was used to measure the glass transition temperature (*T*_g_). The anti-oxidant activity was assessed by the DPPH assay.^16^ ATR-FTIR spectra were recorded using a Spectrum One spectrophotometer (PerkinElmer, Monza, Italy). For this purpose, the sample under examination (∼2.0 mg) was placed on a diamond crystal mounted in an ATR cell (PerkinElmer, Monza, Italy). The spectra were recorded at 4 cm^–1^ resolution and 128 scans were collected over the wavenumber region 4000–650 cm^–1^.

### 2.3 FLV-Eluting NP

#### 2.3.1 NP Preparation

To prepare a FLV-eluting NPs, the drug was dissolved in the aqueous phase to get the theoretical concentration of 25% w/w. An aliquot of 0.2 mL was emulsified with 1 mL of polymer solution by an ultrasonic sonotrode with a tip diameter of 2 mm at 60% amplitude (Hielscher Ultrasound Technology, G) in an ice bath. The W/O emulsion was dispersed in 2.5 mL of aqueous phase under sonication in an ice bath. Then, it was diluted with 8 mL of 0.3% PVA solution, and the temperature was slowly risen to 30 °C to favor the solvent evaporation. The loaded NPs were collected by centrifugation at 11,000 rpm for 30 min at 4 ± 1 °C (Universal 30 RF, Hettich GmbH & Co., G) and suspended in Milli-Q water.

#### 2.3.2 Drug Content

About 2 mg exactly weighed of the freeze-dried NPs were incubated in 10 mL of mobile phase to extract FLV. Then, the solutions were filtered through a membrane filter (pore size = 0.45 μm, Millipore, I) before HPLC measurements. The experimental FLV loading (as %) and the encapsulation efficiency (EE, %) were calculated. All operations were carried out away from light.

#### 2.3.3 In Vitro FLV Release

The drug release studies from NPs were conducted in triplicates using a dialysis membrane made of regenerated cellulose with a MWCO = 8–10 kDa (Float-A-Lyzer G2, ThermoFisher, I) loading 5 mL of nanosuspension. The dialysis tube was immersed in 19 mL of pH 7.4 PBS at 37 ± 1 °C under magnetic stirring at 1600 rpm. At predetermined time points, 200 μL of the release medium was withdrawn and replaced with fresh buffer. At the end of the experiment, the nanosuspension was centrifuged twice at 10,000 rpm for 30 min; the supernatants withdrawn, and the pellet was dissolved in the mobile phase to extract the remaining drug. The diffusion profile of the pure FLV was also obtained using the same testing conditions.

#### 2.3.4 HPLC Method

The drug quantification was carried out using an HPLC (HP1100 series, Agilent, UK), equipped with a quaternary pump, an auto-sampler, a thermostated column compartment at 25.0 ± 0.1 °C, and a DAD-UV detector. An aliquot of 10 μL was eluted through a C18 column (Phenomenex Luna, LC 5 μm, 150 × 46 mm) using a mixture of acetonitrile/pH = 3.0 phosphate buffer (60:40% v/v) at the flow rate of 1.2 mL/min. The detection of FLV was performed at 230 nm. Calibration curve in the range of 1–50 μg/mL (*R*^2^ > 0.99).

### 2.4 Fluorescent NPs

Placebo and fluorescent NPs were prepared by nanoprecipitation using a lab-scale membrane emulsification system (Micropore LDC-1, Micropore Technologies Ltd, UK) equipped with a stirred cell. Briefly, a solution constituted of 1% PLGA or *g*-AA-PLGA in acetone (2 mL) was loaded into a disposable syringe connected to a syringe infusion pump (Aladdin AL300, UK). The organic phase was pushed at the rate of 0.1 mL/min through the pores of a precision engineered stainless steel membrane into 25 mL of Milli-Q water, while a paddle, positioned above the membrane and stirred at 1000 rpm, induced a shear force which results in droplet detachment. The dispersion was left under stirring for 2 h to evaporate the solvent. FITC-NPs were similarly prepared using 10% w/w of FITC-PLGA.^32^ In the case of DIL, the polymer solution was mixed with 0.01% DIL-in methanol in volume ratio 1:0.0025 before injection.

NPs were characterized in terms of size and zeta potential using a Zetasizer Nano ZS DLS instrument (Malvern Instruments, UK) at 25.0 ± 0.1 °C, and DIL-loaded NPs were also characterized at 565 nm long-pass filter both in scattering and fluorescent modes using nanoparticle tracking analysis (NTA, Malvern Instruments, UK), and their stability, expressed in terms of size distribution and concentration, was monitored over a 10 day period.

### 2.5 Electron-Dense NPs

The Ag–PVP probe was prepared as follows: 50 mg of silver nitrate was dissolved in 10 mL of distilled water; then, 1 g of PVP was added and the solution was stirred overnight at room temperature. The obtained solution was introduced into dialysis tubes (molecular weight cutoff, MWCO: 12–14,000 Da, supplied by Medicell International Ltd, London, UK) and dipped into a glass vessel containing distilled water at room temperature for 24 h with four changes of water. Finally, the resulting solution was frozen and dried with a Freeze Dryer Micro Modulo (Edwards, UK).

The silver content of the prepared Ag–PVP probe was evaluated by digesting the sample with 70% HNO_3_ in a microwave at 150 °C for 10 min.^33^ Then, the digested sample was diluted with distilled water to reduce the HNO_3_ concentration to 3.5%. The silver concentration was evaluated by inductively coupled plasma mass spectroscopy (ICP–MS) (PerkinElmer Elan DRCII).

Nanosuspension was obtained as described in Section 2.4 using an organic phase constituted of PLGA or *g*-CA-PLGA and Ag–PVP in the ratio from 1:9 to 3:7. The morphology of electrondense NPs was assessed by TEM.

### 2.6 Cells

The human SMC line was purchased from ATCC, USA (PCS-100-012) and was used between the fourth and the seventh passage. The culture medium used is ATCC Vascular Cell Basal Medium, added with 500 μL of ascorbic acid, 500 μL of rh EGF, 500 μL of rh insulin and rh FGF-b, 25 mL of glutamine, 25 mL of FBS (ATCC Vascular Smooth Muscle Growth Kit), and 5 mL of penicillin–streptomycin 100× (Euroclone, Milan, I).

The human endothelial EA.hy926 cell (EHEC) line (ATCC CRL-2922, USA) was cultured in high glucose Dulbecco’s modified Eagle’s medium (DMEM; EuroClone, I) supplemented with 10% fetal bovine serum (FBS), 2 mM l-glutamine, 100 U/mL penicillin, 100 μg/mL streptomycin, and 2% HAT supplement (Sigma-Aldrich, I).

The human monocyte-derived macrophages were isolated from the blood of healthy volunteers and cultured in with DMEM containing 10% human AB serum and insulin (8 μg/mL).

### 2.7 MTT Assay

Cellular toxicity caused by *g*-AA-PLGA NP was assessed both by measuring cellular protein content and by using the 3-(4,5-dimethylthiazol-2-yl)-2,5-diphenyltetrazolium bromide (MTT) colorimetric assay which relies on the ability of viable cells to actively metabolize the dye. Briefly, after incubation with NP (100 μg/mL) for 24 h, cells were washed with PBS and MTT (Sigma-Aldrich, I) was added at a concentration of 10 μg/mL in the culture medium. Following 90 min of incubation, the supernatants were discarded, the intracellular formazan precipitates were solubilized by the addition of 100% DMSO and plates placed on a plate shaker for 10 min. Absorbance was evaluated at 620 nm. A 4% solution of Triton was used as a positive cytotoxic control.

### 2.8 Cellular Uptake of NP

Cellular uptake of fluorescent PLGA, *g*-CA-PLGA, and *g*-RV-PLGA NP was evaluated in human SMCs, macrophages, and endothelial cells. Briefly, cells were seeded at the cellular density of 30,000 cells/well in a 96-well plate. After reaching the confluence, cells were washed with PBS and the culture medium with 0.2% EFAF containing the fluorescent NPs at a final concentration of 100 μg/mL was added. Incubation times were 0.5, 2, and 4 h for macrophages and 4, 16, and 24 h for SMCs and endothelial cells. After the incubation period, plates were cooled with ice, media were removed, and cells were washed three times with cold PBS and fixed with methanol for 20 min. After methanol removal, cells were washed again three times with PBS. Culture medium without NPs was used as the negative control. A microplate reader (Wallac 1420 Victor^2^ Microplate Reader, PerkinElmer, I) was used to measure the fluorescence intensity from up-taken NPs in each well at 25.0 ± 0.1 °C, with lamp filter and emission filter at 535 and at 485 nm, respectively. The cellular uptake efficiency was calculated normalizing the observed fluorescence intensity in each well (*I*_OBS_) by the mean fluorescence intensity of the negative control (*I*_NC_), as reported in eq 1
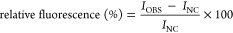
1

Only the samples with a fluorescence higher than the highest fluorescence registered for the negative control were used for the analysis. Results are reported as mean ± SEM (*n* = 6).

### 2.9 TEM Analysis

The uptake of NPs and their distribution inside the three cell lines were deepened by transmission electron microscopy (TEM) using Ag-NP in order to assure a simpler recognition. Macrophages, endothelial cells, and SMCs were seeded at a cellular density of 300,000 cells in a 35 mm diameter Petri dish and incubated for 96 h. Cells were then washed with PBS and treated for 4 h with placebo *g-*CA-PLGA NP diluted with MEM + 0.2% EFAF (MEM + 0.2% EFAF without NP was also used as a negative control). After washing with PBS, cells were detached with trypsin and centrifuged at 1500 rpm for 15 min. Pellets were washed with 0.1 M sodium cacodylate buffer at pH 7.3 and centrifuged twice, as previously reported. Pellets were fixed overnight in a solution containing 2% of freshly prepared paraformaldehyde and 2% glutaraldehyde in 0.1 M sodium cacodylate buffer at pH 7.4. Samples were rinsed twice in the same cacodylate buffer for 30 min and post-fixed in 1% osmium tetroxide in a cacodylate buffer 0.1 M at 0 °C for 90 min. Pellets were then washed in distilled water followed by a staining with 2% aqueous uranyl acetate, dehydrated in a graded acetone series, and embedded in an Epon-Araldite resin. Ultrathin sections were cut by a Leica Supernova ultramicrotome (Reichert Ultracut, Wien, Austria) and counterstained with lead citrate. TEM was performed with a Zeiss EM10 electron microscope (Carl Zeiss, Oberkochen, Germany).

### 2.10 Confocal Analysis

Human SMCs were seeded at a density of 10^5^ cells *per* well in 200 μL of DMEM media on μ-slide eight-well glass-bottom dishes (ibidi, GER). After 24 h, ∼3 × 10^9^ DIL-labeled NPs (PLGA or *g-*CA-PLGA) were added in each well and cells were incubated for up to 24 h. Next, the cells were washed three times with PBS to remove NP not internalized, fixed with paraformaldehyde 4% at room temperature for 10 s, washed three times with PBS, and stained at room temperature for 5 min with a solution of CellMask deep red (Invitrogen, US) and Hoechst 33342 (Invitrogen, US), both at a final concentration of 2 μg/ml. After final washing, the cells were imaged using a confocal microscope (Leica Stellaris, GER) with either a 40× air or 63× glycerol objective. Quantification of DIL fluorescence *per* cell and *per* field of view were measured by processing raw data with NIS-Elements v.5.2 digital imaging analysis software (Nikon Instruments, JAP) for segmentation and precise quantification, implementing the general-analysis tool-box.

### 2.11 Evaluation of Cell Proliferation and Migration

In order to evaluate the effect of free and NP encapsulated FLV on cell proliferation, SMCs and endothelial cells were seeded at 5000 cells/well in a 12-well plate until confluence. Cells were treated with FLV dissolved in ethanol and FLV-NP at the drug concentration of 1, 2, 4, 6, and 10 μM using ethanol and placebo NP as negative controls. After the removal of the treatments, cells were detached with trypsin and counted with a Coulter Counter (Z1 Beckman Coulter, I), calibrated at the threshold of 6.8 μm. Results are reported as mean ± SEM (*n* = 6).

For the evaluation of cell migration, the in vitro scratch wound method was used. Human SMCs and endothelial cells were seeded at the density of 5000 cells/well in a 12-well plate and incubated in media until confluence was reached. After a washing step with PBS, fresh medium was added before the treatment. A wound in cellular monolayers was made by using a sterile disposable tip and detached cells were removed by washing with PBS. Then, cells were incubated for 24 h with increasing concentrations (2, 4, and 10 μM) of FLV-NP and the matching concentrations of either placebo NP or FLV solution in ethanol (vehicle). Cell directional migration was evaluated by measuring the cell ability to reclose the wounded area by using a phase-contrast microscope and photographed using a digital camera (Axiovert 200, Zeiss, G). Images were acquired after 24, 48, 72, and 144 h. For quantitative representation of the results, the percentage of reclosure of the scratched area in the monolayer was determined by using AxioVision software (Zeiss, USA). Results are reported as mean ± SEM (*n* = 6).

### 2.12 Statistical Analysis

One-way and two-way analysis of variance (ANOVA) followed by Tukey’s test as post ANOVA means comparisons were performed using OriginPro 2015 (OriginLab Corporation, USA), considering the different types of polymer and the time of incubation with cells as factors. Differences were considered statistically significant at a level of α = 0.05. Outliers were discarded according to Dixon’s T-test.

## 3 Results and Discussion

### 3.1 Synthesis and Characterization of *g-*AA-PLGA and Ag–PVP

In the present study, anti-oxidant-grafted PLGA (*g-*AA-PLGA) was prepared according to a free radical grafting approach involving two main reaction steps and the use of an ascorbic acid/hydrogen peroxide redox pair.

In the first step of the reaction, the initiator system generates radical species that react with PLGA leading to the formation of macroradicals; the following step involves the insertion of the anti-oxidant molecules, such as CA or RV, onto the polymeric chain through the formation of covalent bonds. In a previous study,^17^ it was hypothesized that hydroxyl radicals, resulting from the oxidation of ascorbic acid in the presence of hydrogen peroxide, activated PLGA initiating the grafting reaction. A further study highlighted a different reaction mechanism mediated by ascorbate radicals (Asc^•–^).^34^

**Figure 1:**
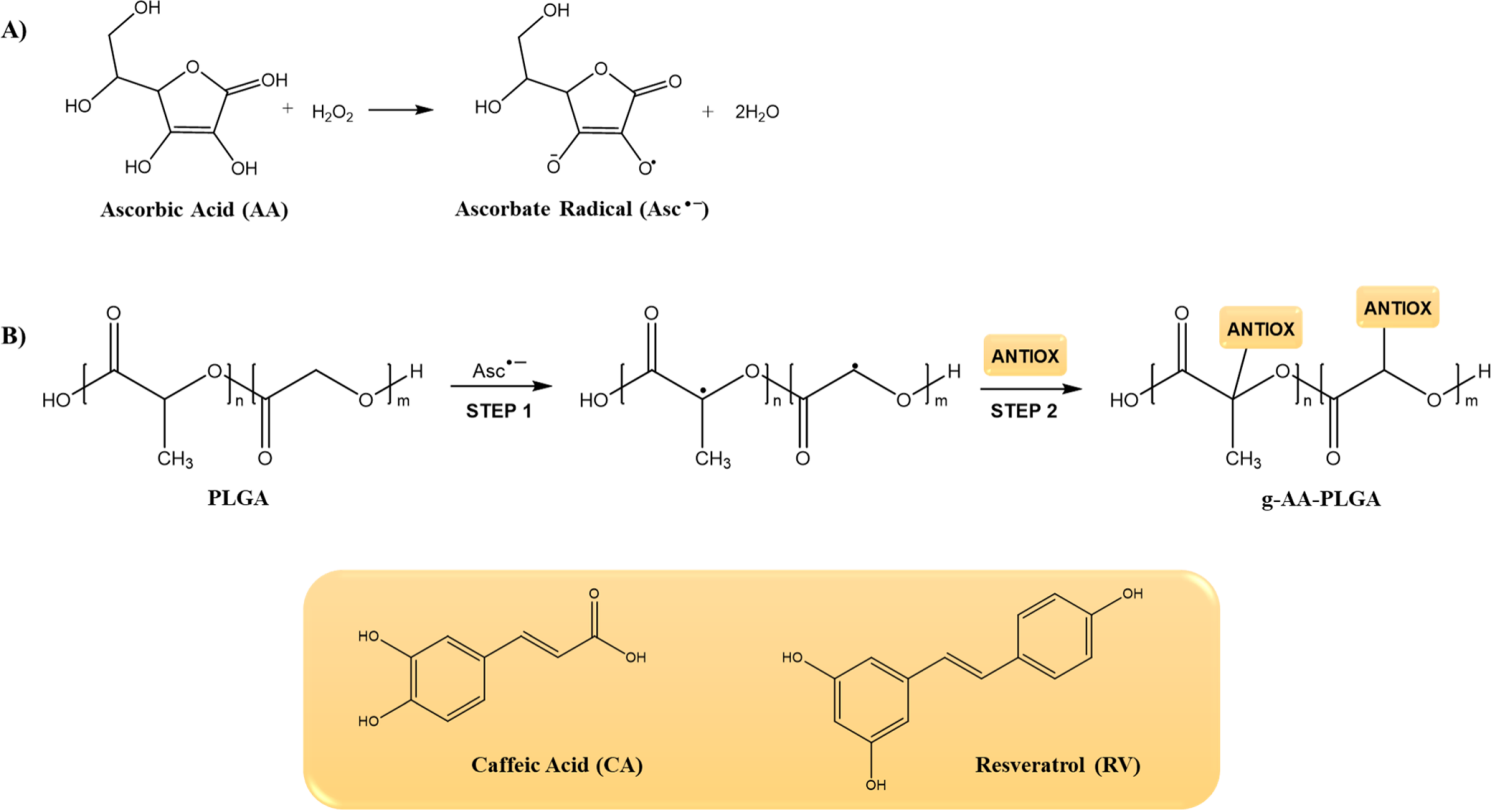
(A) Ascorbic acid/hydrogen peroxide reaction mechanism and (B) reaction scheme for the synthesis of *g-*AA-PLGA.

Ascorbic acid (AA), indeed, is susceptible to autoxidation in water solution due to the presence of two ionizable hydroxyl groups, with the generation of ascorbate radicals. In detail, the dissociation of 3-OH and 2-OH leads to the formation of ascorbate monoanion (AscH^–^) and ascorbate dianion (Asc^2–^). The first one, which is an excellent reducing agent, undergoes two successive one-electron oxidations producing ascorbate radical and, finally, dehydroascorbic acid. The AA autoxidation is accelerated by the presence of hydrogen peroxide with a higher Asc^•–^ production according to the mechanism reported in Figure 1A.^35^

The so formed ascorbate radicals react with PLGA to abstract hydrogens on carbons alpha to carbonyl groups forming macroradicals (Figure 1B). The subsequent step involves the reaction of the macroradicals active sites with the ortho- and para-positions relative to the hydroxyl groups of the anti-oxidant compounds leading to the insertion of these molecules onto the PLGA backbone.^36,37^

The main features of PLGA and *g-*AA-PLGA raw materials are summarized in Table 1. The data confirmed the suitability of the synthetic strategy, which permits the conjugation of CA or RV on the copolymer backbone without a consistent modification of *M*_w_ and *T*_g_. Furthermore, the comparison of the data with those obtained in a previous work confirmed the good reproducibility of this mild grafting technique.^17^ The DPPH assay was conducted to evaluate whether RV and CA retained their anti-oxidant capacity after grafting on the PLGA backbone. As expected, the control polymer did not show any anti-oxidant activity, while *g-*CA-PLGA exhibited a higher radical scavenging activity with respect to *g-*RV-PLGA (Table 1).

**Table 1:** Physico-Chemical Features of *g-*CA-PLGA and *g-*RV-PLGA and Their Anti-oxidant Properties Expressed as DPPH Radical Inhibition (%)

				DPPH radical inhibition (%)			
polymer	*M*_w_ (KDa)	PI	*T*_g_ (°C)	1 h	2 h	3 h	24 h
PLGA	20.4	1.52	35.8	0 ± 0.4	0 ± 0.4	0 ± 0.5	0 ± 0.3
*g-*CA-PLGA	18.4	1.65	34.5	90 ± 0.4	92 ± 0.7	93 ± 0.6	97 ± 0.6
*g-*RV-PLGA	18.5	1.38	34.8	27 ± 0.8	32 ± 0.4	37 ± 0.3	56 ± 0.5

The Ag–PVP probe, obtained by the chelation of silver by PVP oxygen and nitrogen atoms, presented a monomodal distribution with a hydrodynamic diameter (expressed as volume) of about 12 nm. Inductively coupled plasma mass spectrometry (ICP–MS) was employed to evaluate the silver content of Ag–PVP and the performed analysis revealed a silver content equal to 11.8 mg/g. This complex was designed because Ag guarantees an electron-dense signal in TEM analyses of the biological sample and PVP, as a hydrophilic portion, assures a good dispersibility in water and methylene chloride and a suitable stability of the dispersed system NPs.

### 3.2 Characterization of NPs

The different physico-chemical characteristics of NP components required the adoption of different preparation methods. In all cases, the experimental set up allowed the obtainment of monodispersed NPs with similar hydrodynamic diameters (*D*_H_) and negatively charged with zeta potential of about −30 mV (Table 2).

**Table 2:** Main Features of Different Types of NPs

form. ID	main constituent(s)	FLV (%)	D_H_ (nm)	PdI	ζ (mV)
PLGA-NP	PLGA		186 ± 1	0.061 ± 0.016	–32.4 ± 0.6
CA-NP	*g*-CA-PLGA		171 ± 2	0.082 ± 0.020	–30.4 ± 1.0
RV-NP	*g*-RV-PLGA		193 ± 3	0.064 ± 0.016	–32.5 ± 0.6
F-PLGA-NP	PLGA/FITC-PLGA (10%)		172 ± 1	0.090 ± 0.017	–29.7 ± 0.4
F-CA-NP	*g*-CA-PLGA/FITC-PLGA (10%)		176 ± 1	0.087 ± 0.015	–30.0 ± 0.2
F-RV-NP	*g*-RV-PLGA/FITC-PLGA (10%)		185 ± 1	0.083 ± 0.013	–29.4 ± 0.6
Ag-PLGA-NP	PLGA/Ag–PVP (20%)		172 ± 1	0.143 ± 0.024	–29.1 ± 0.9
Ag-CA-NP	*g*-CA-PLGA/Ag–PVP (20%)		230 ± 3	0.253 ± 0.008	–17.4 ± 0.3
FLV-PLGA-NP	PLGA	4.5 ± 0.7	175 ± 3	0.125 ± 0.045	–33.1 ± 0.3
FLV-NP	*g*-CA-PLGA	10.6 ± 1.0	185 ± 1	0.133 ± 0.028	–28.4 ± 2.1
FLV-RV-NP	*g*-RV-PLGA	9.1 ± 2.0	180 ± 2	0.145 ± 0.018	–29.7 ± 1.2

**Figure 2:**
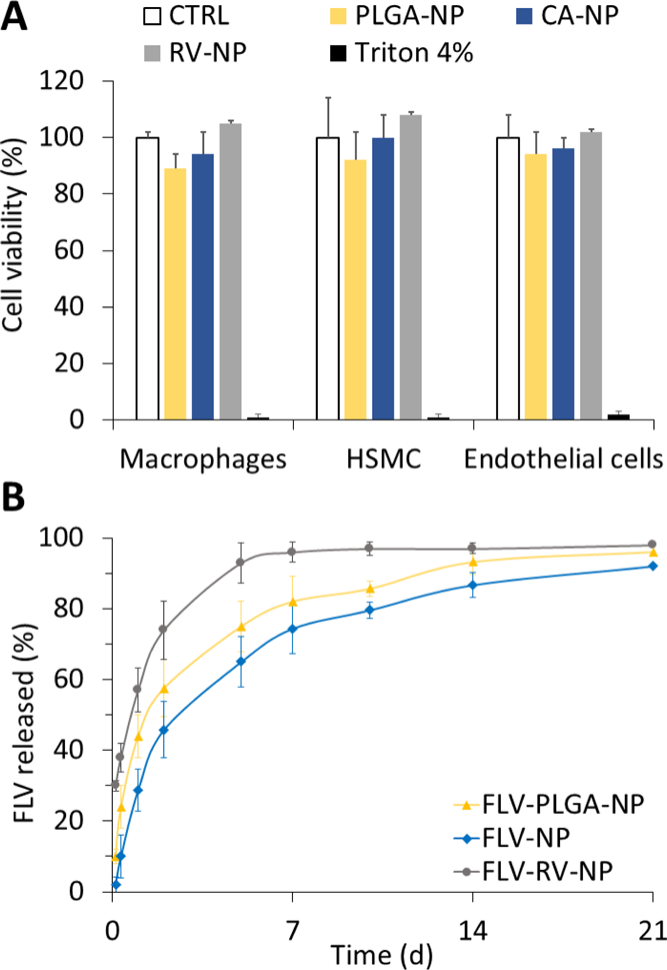
(A) Cell viability after 24 h of incubation with the NP. (B) In vitro release profiles of FLV from NP made of PLGA (FLV-PLGA-NP), *g*-CA-PLGA (FLV-NP), and *g*-RV-PLGA (FLV-RV-NP).

Next, we assessed the biocompatibility of placebo NP on macrophages, endothelial cells, and SMCs representatives of the main cell types present in the vessel wall. Overall, the grafted polymers were well tolerated by all cell lines, as not-significant differences in cell viability were observed compared to control culture medium alone (control, Figure 2A). This suggests that the anti-oxidant-based grafting does not affect the in vitro cytotoxicity of PLGA.

The further modification of the preparation method required to encapsulate highly water-soluble FLV into polymeric NP did not affect NP main features. As expected by previous experience with ovalbumin,^17^ the use of *g-*AA-PLGA allowed the loading of a double amount of FLV (Table 2).

However, the drug release profile obtained with the two grafted copolymers was different. FLV-RV-NP gave a high burst effect because about 30% FLV was released at early data points, and the plateau was reached in about 7 days (Figure 2B). As in the previous case, the FLV burst effect was higher for PLGA NP (i.e., about 10%) compared to the *g-*CA-PLGA, which was considered negligible. Nevertheless, the drug release rate constants, calculated according to Higuchi’s equation (*K*), overlapped (FLV-PLGA-NP: *K* = 0.341 ± 0.010 d^–1^; FLV-NP: *K* = 0.337 ± 0.012 d^–1^) and a prolonged release of FLV was assured beyond 3 weeks for both formulations.

To evaluate the internalization by the different cell types present in the arterial wall, NPs made of grafted polymers and with different features were prepared. Specifically, NPs loaded with FITC or DIL to be used for fluorescence imaging, or electrondense NPs to be employed for TEM assessments. NPs containing 10% w/w FITC-PLGA maintained the same features of the placebo NP (Table 2) and were, therefore, used to estimate the cellular uptake in the three cell lines. Nevertheless, the FITC signal is subject to high quenching and, therefore, NPs loaded with DIL were preferred for confocal imaging. In this latter case, some preliminary experiments were carried out to optimize the probe/copolymer ratio and the selection of organic solvent(s) to obtain NPs by using the solvent displacement method. To avoid aggregation, the solution containing 0.01% DIL in methanol was mixed to the PLGA solution in acetone. The slight reduction in surface charge from −30 mV to about −4 mV in DIL-PLGA-NP was due to the probe adsorption on the NP surface. These data were also supported by the slight increase of particle size distribution derived from NTA analyses after the activation of a 565 nm long-pass filter for green laser. Indeed, the non-fluorescent PLGA-NP showed *D*_50_ = 117 ± 2 nm and *D*_90_ = 174 ± 11 nm which shifted toward *D*_50_ = 135 ± 2 nm and *D*_90_ = 208 ± 3 nm in the case of the fluorescent PLGA-NP. These values did not undergo any variations over a 10 day period of time, (*t*_0_: 6.85 × 10^10^ ± 2.86 × 10^8^ particle/mL; *t*_10_: 7.03 × 10^10^ ± 8.40 × 10^8^ particle/mL; *D*_50_ = 136 ± 2 nm; *D*_90_ = 214 ± 1 nm), confirming the stability of the labeling. Similarly, DIL-CA-NP presented a monotonic dispersion (*D*_50_ = 150 ± 5 nm and *D*_90_ = 226 ± 212 nm) and a similar concentration (3.34 × 10^10^ ± 1.23 × 10^9^ particles/mL) which did not significantly change over 10 days of storage at 4 °C.

**Figure 3:**
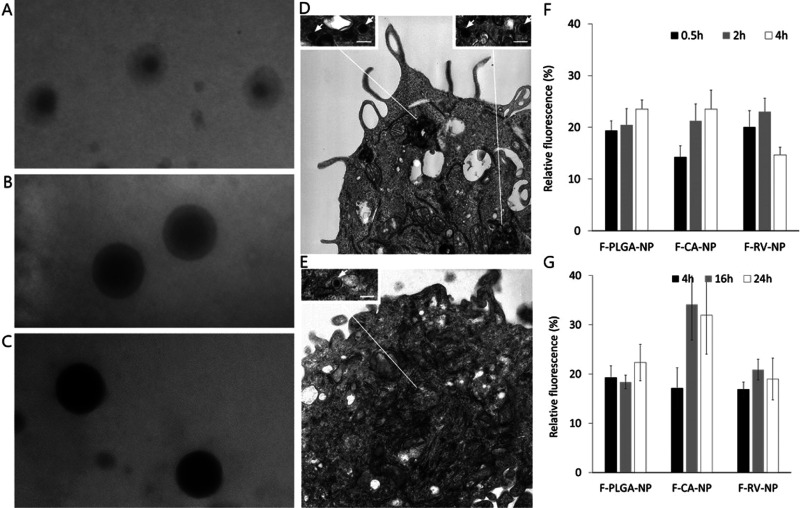
Ag–PVP conjugate loading in NPs ranged from 10 to 30% w/w without influencing the hydrodynamic diameter and PDI (*D*_H_ ∼ 280 nm). To optimize the conjugate content, NPs were observed by TEM. At 10% loading, the electrodense core was not uniformly defined (A); while at the highest content, the entire NP appeared dark (C). Only at 20% loading, it was possible to clearly detect an electrodense core surrounded by a PLGA wall (B). This peculiar structure also allowed discriminating among NPs if Ag–PVP-loaded NPs were mixed by blank NPs at a 1:1 ratio. Panel (D) reports a representative TE micrograph showing a macrophage containing within the cytoplasm a large phagosome. Arrows point at some NPs between the ingested material. Scale bar: 100 nm. Panel (E) shows a typical TE micrograph of an endothelial cell showing a single NP particle (arrow) dispersed within the cytoplasm. Scale bar: 100 nm. Finally, panels (F,G) report the uptaken PLGA-NP, F-CA-NP, and F-RV-NP in macrophages and endothelial cells, respectively.

Finally, electron-dense NPs (Ag-PLGA-NP and Ag-CA-NP) were designed to properly distinguish the polymeric constructs from cellular organelles in TE microphotographs. The loading of 20% Ag–PVP was considered optimal because NP presented a well-defined core surrounded by a PLGA wall (Figure 3B). Conversely, NP with lower Ag content resulted inhomogeneous with a pale core (Figure 3A), and those loaded by the greatest amount resulted completely electrodense (Figure 3C).

### 3.3 . Uptake of Placebo NP

We determined the uptake of NPs made of different copolymers by macrophages, SMCs, and endothelial cells to provide provisional data on the amount of NPs which could be potentially retained in the vessel wall after administration by DEB. We also included the contribution of NPs adsorbed on the cell surface and entrapped in the extracellular space which also can contribute to the availability of FLV because they assure the release of the drug over a prolonged period of time. As expected, NPs were taken up faster by macrophages compared to the other cell lines, as intracellular fluorescence was already detectable after only 30 min of exposure (Figure 3F). Moreover, the grafting of CA or RV on the PLGA backbone did not influence the uptake process, independent of the incubation time (two-way ANOVA, *p* > 0.05) (Figure 3F).

In the case of endothelial cells, after 16 h of exposure to the NPs, the fluorescence in the cells treated with F-CA-NP resulted significantly higher than in those treated with F-PLGA-NP or F-RV-NP [one-way ANOVA followed by Tukey’s test, F-CA-NP vs F-PLGA-NP (*p* = 0.028) and F-CA-NP vs F-RV-NP (*p* = 0.032), Figure 3G].

**Figure 4:**
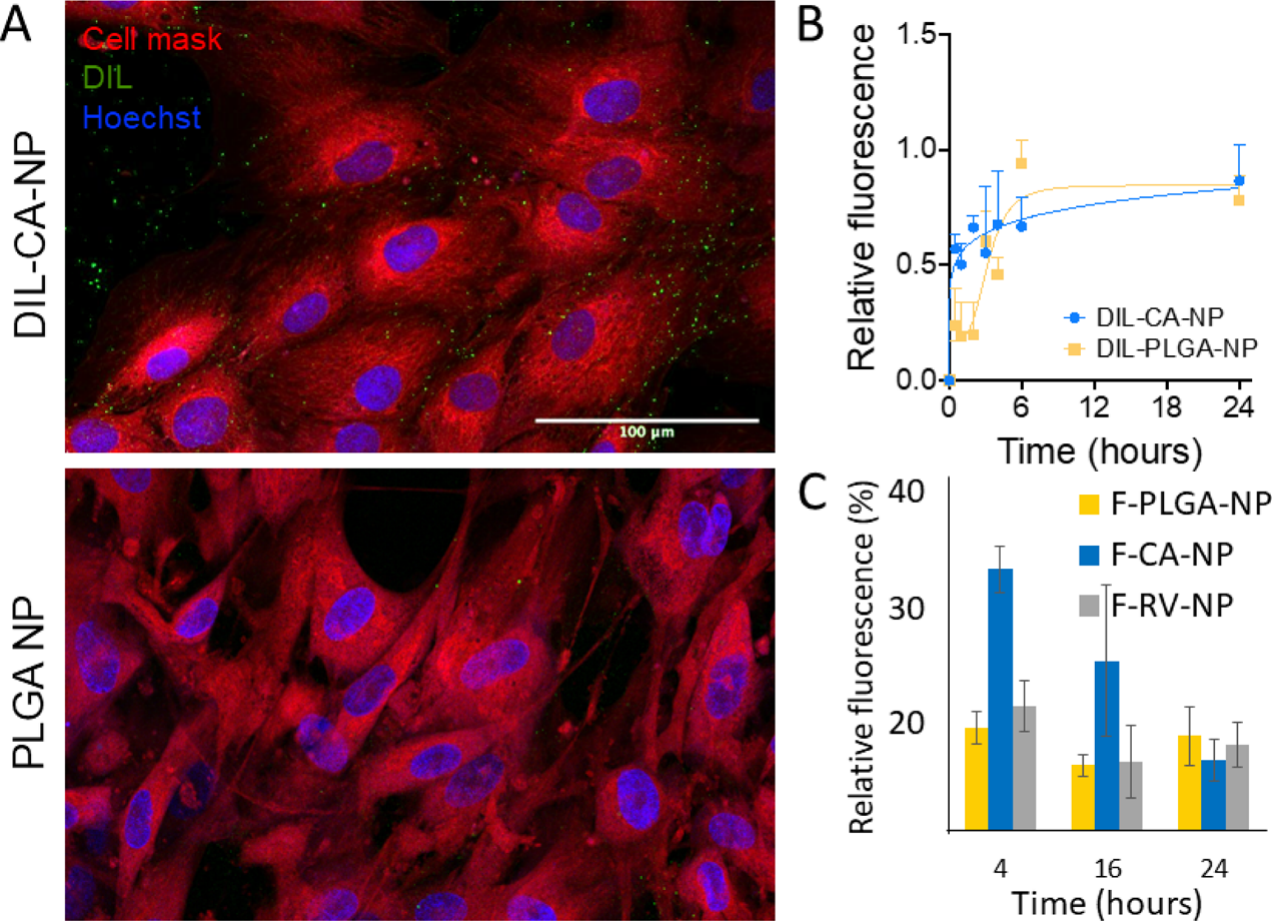
Panel (A) shows representative images of SMCs incubated for 1 h with either DIL-CA-NP or DIL-PLGA-NP and imaged by confocal microscopy. Panel (B) reports quantification of the intracellular fluorescence measured on single cells over 24 h (mean ± SD). Panel (C) is related to the uptake of PLGA-NP, F-CA-NP, and F-RV-NP in SMCs at different time points.

On the contrary, SMCs took up F-CA-NP more rapidly than the other two formulations, (one-way ANOVA followed by Tukey’s test, F-CA-NP vs PLGA-NP *p* = 0.002, F-CA-NP vs F-RV-NP *p* = 0.004 and F-RV-NP vs PLGA-NP *p* = 0.984, Figure 4A). Afterward, a significant decrease in the relative fluorescence (%) was observed only for F-CA-NP. Considering that no cytotoxic effects were detected over a 24 h-period, the reduced ability to sustain the NP uptake can be due to the beginning of exocytosis, as already reported in literature.^38^ Conversely, no statistical differences were found in NP concentration over time for the other two formulations (Figure 4A). Considering (i) the poor ability of *g-*RV-PLGA to control the FLV release and that (ii) no statistical differences in the uptake of PLGA-NP were observed in all cell lines, this polymer was discarded from further evaluations.

The deeper insight using TEM revealed that the different distributions of NPs in the cell lines were independent of the polymer. The most distinctive feature is the presence of endocytotic vesicles, lysosomes, and phagolysosomes within the cytoplasm of macrophages (Figure 3D), which is a fingerprint of such professional phagocytes. In this case, we evidenced the presence of some internalized NPs interposed between other ingested material (Figure 3D). The ability of the other two cell lines to uptake NPs appears different because single Ag-CA-NP particles were found to be dispersed in the cytoplasm, as exemplified in Figure 3E for endothelial cells.

We decided to further investigate the internalization of both DIL-PLGA-NP and DIL-CA-NP by SMCs because FLV should inhibit their proliferation which is one of the main causes of restenosis. SMCs were cultured with both NPs at different incubation times, ranging from 0.5 to 24 h, and then imaged by confocal microscopy (Figure 4A).

The results demonstrate that NPs were rapidly internalized and accumulated inside the cells as a function of time, regardless of the presence of the CA on the PLGA backbone. Overall, the fluorescent signal observed was punctiform and widely distributed across the cell cytoplasm. We further performed image analysis on single cells to quantify the relative fluorescence associated to the dye. The measurements revealed that, over the first 6 h of incubation, DIL-CA-NP had a faster dynamic of internalization compared to the pristine preparation (Figure 4B). The quantification of the relative intracellular fluorescence suggests that the presence of CA in *g-*CA-PLGA could favor the initial binding of the NP to the SMCs promoting a rapid internalization, as verified in the set of experiments performed using FITC PLGA conjugate as a probe (Figure 4C).

Thus, on the bases of all data, *g-*CA-PLGA was selected as the most promising material for the in vitro studies on cell proliferation.

### 3.4 Effect of FLV-NP on Human SMC and Endothelial Cell Proliferation and Migration

To test the efficacy of NPs loaded with FLV in inhibiting SMC proliferation, human SMCs and endothelial cells were incubated with increasing concentration of FLV-NP for 24 h and with the matching concentrations of either the placebo NP or a FLV solution.

**Figure 5:**
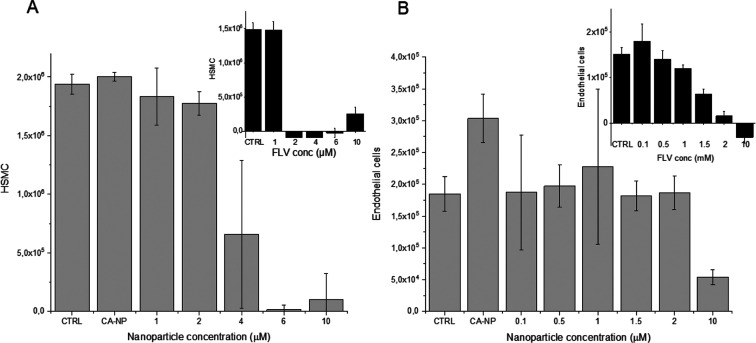
Effect of FLV-NP and fluvastatin (insert) on (A) human SMC proliferation and (B) endothelial cell proliferation.

As expected,^39^ the drug alone inhibited SMC proliferation in a statistically significant manner starting at concentrations higher than 1.2 μM (at 2 μM cell proliferation was inhibited by 100%) (Figure 5A).

FLV-NP were also effective in inhibiting SMC proliferation: at 4 and 6 μM, cell proliferation was reduced by 50 and 100%, respectively (Figure 5B). This demonstrates that NPs efficiently delivered the encapsulated drug to cells and that the drug was still pharmacologically active and able to affect cell behavior.

In endothelial cells, the addition of FLV resulted in a concentration-dependent and statistically significant reduction of cell proliferation, with the maximal inhibitory effect achieved at a concentration of 1.5 μM (Figure 5C). On the contrary, the incubation with FLV-NP did not affect endothelial cell proliferation, except for the highest concentration tested (10 μM, Figure 5D). Surprisingly, the addition of the placebo NP stimulated the endothelial cell proliferation by about 50% (Figure 5D).

Finally, the effects of FLV-NP on cell migration were analyzed by using the directional migration assay.^40^

**Figure 6:**
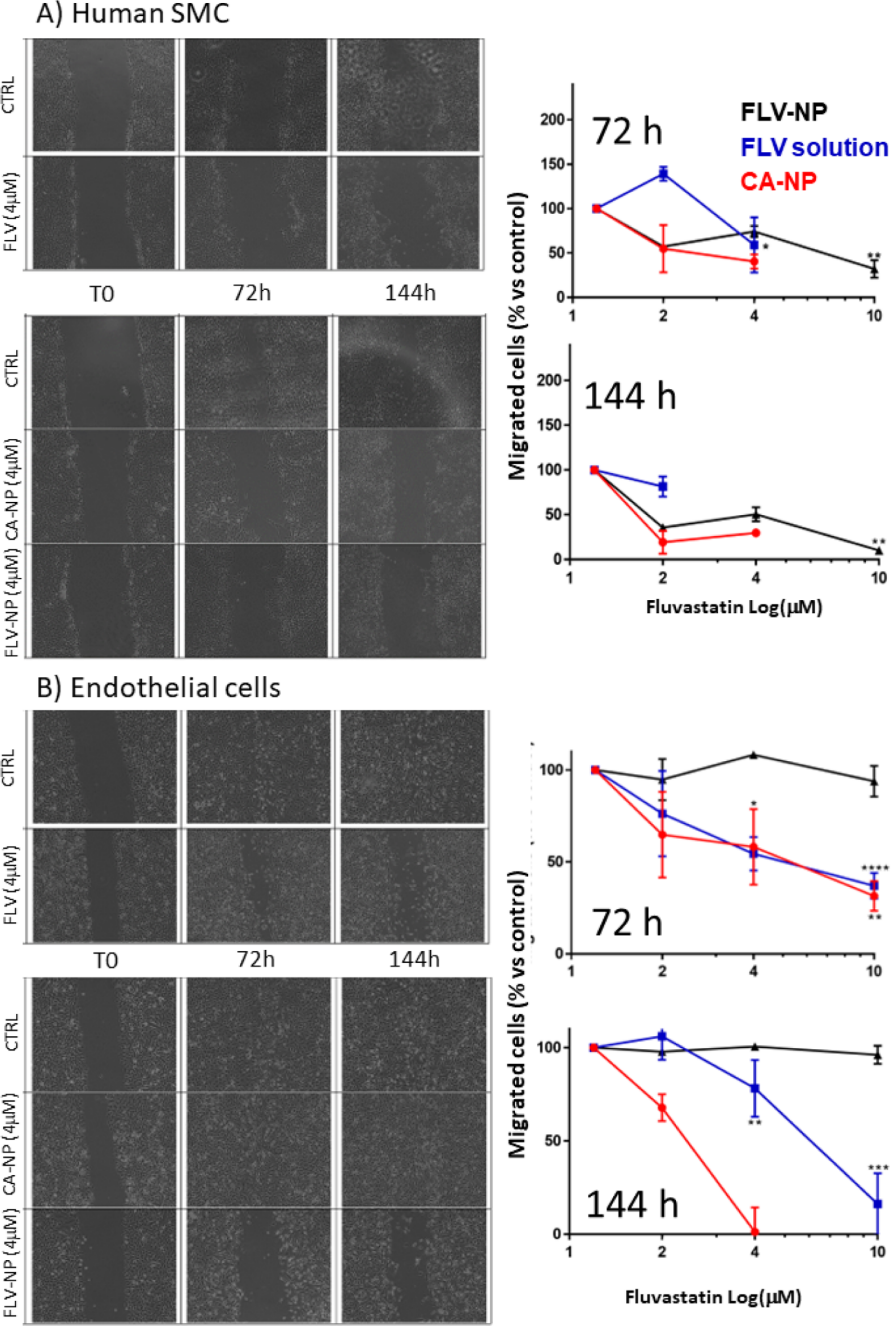
Effect of fluvastatin and FLU-NP on directional migration of (A) human SMCs and (B) endothelial cells at different time points. Black: CA-NP; red: FLV solution; Blu: FLV-NP (*p < 0.05; **p < 0.01; ***p < 0.001; and ****p < 0.0001 vs CTRL).

The drug alone and the FLV-NP reduced human SMC directional migration in a concentration and time-dependent manner (up to 80% inhibition at the longest time-point tested, Figure 6). Enhanced migration of human SMCs with an almost completed wound closure was evident after 144 h when cells were treated with the vehicle alone, indicating that they preserved the motility in our experimental conditions. Similar results were obtained after the treatment with placebo NP. When cells were treated with FLV alone or with FLV-NP, a significant area of the wound remained not covered.

The addition of FLV alone and of FLV-NP also reduced endothelial cell migration in a concentration- and time-dependent manner. The placebo NP did not affect this process (data not shown). After the treatments with vehicle, culture medium (negative controls), or CA-NP, the wound areas were completely reclosed, while in the presence of FLV alone or FLV-NP, the wound areas were still uncovered at the latest time point, particularly evident in the case of FLV-NP.

All together, the data show that *g-*CA-PLGA may assure a prolonged release of FLV in a pharmacologically effective form. Indeed, upon administration of NP in the injured vessel, the drug release can be assured by two different mechanisms: the diffusion from NP adsorbed in the extracellular matrix and the cellular uptake. Indeed, the data demonstrated that the FLV-NPs effectively enter into SMC, endothelial cells, and macrophages. In particular, a concentration- and time-dependent inhibition of human SMC proliferation was observed after treatment with FLV-NP, with a 50% inhibition at 4 μM and the maximal effect at 6 μM. As expected, this effect is achieved at a FLV-NP concentration slightly higher than what we obtained by the direct addition of FLV to cells that completely abolished SMC proliferation at a concentration of 2 μM. In the case of endothelial cells, FLV blocked the proliferation in a concentration-dependent manner. Conversely, when the drug was delivered by FLV-NP, no inhibitory effect on the endothelial cells was observed, except at the highest concentration tested (10 μM) which most probably highlights a toxic effect on the cell monolayer. Of note, the addition of placebo NP resulted in a stimulation of endothelial cells proliferation by about 30%, while no effect was observed on SMCs. All together, these data suggested that the encapsulation of FLV in *g-*CA-PLGA NP seems to improve the selectivity of the drug toward SMCs rather than endothelial cells.

Regarding the effects on proliferation, FLV-NP inhibited the directional migration of human SMCs in a concentration-dependent manner, achieving its maximal inhibitory effect already after 48 h of incubation at the highest concentration tested (data not shown). At later time points (72 and 144 h), at the 2 μM concentration, the FLV-NP resulted more efficacious in inhibiting the SMC migration with respect to the FLV solution (Figure 6A), confirming the benefits of assuring a prolonged release by NP. It should also be underlined that placebo NP became cytotoxic at the highest concentration (CA-NP, Figure 6A), suggesting a synergic effect of *g-*CA-PLGA and FLV. This cytotoxic effect of placebo NP was not detected in the case of endothelial cells, and the ability of FLV-NP to inhibit the directional migration overlapped with that of FLV solution within the 72 h. At the longest time points, the FLV-NP resulted more effective independently of the tested concentration suggesting that the control of the drug release played a key role in endothelial cell migration (Figure 6B).

These results confirm that statins could be used to inhibit SMC-induced restenosis because they inhibit SMC proliferation and migration, as corroborated by literature data as well.^24,28,29^

Moreover, CA-NPs are suitable for delivering, in an effective and rapid manner, a drug to cells, maintaining its pharmacological properties and therapeutic effects. Some examples of statin-loaded NPs are described in the literature. Pitavastatin-containing NPs were incorporated into the eluting stent and reduced in-stent restenosis in an animal model^25^ or intravenously infused, thus inhibiting left ventricular remodeling^40^ or atherosclerotic plaque destabilization.^41^ However, the intravenous injection of NPs systemically delivers the drug to inflammatory cells (mainly monocytes),^40^ thus reducing the chance to reach other targets, such as SMC and endothelial cells.

## 4 Conclusions

Percutaneous transluminal angioplasty, which allows the disruption of the atheroma, is the main solution to open the narrowed or obstructed artery. The main adverse event connected to this procedure is the restenosis, an inflammatory process leading to the re-occlusion of the vessel. Among different techniques proposed to address this issue, the main solution resides in stenting the vessel or, if it is not possible, in locally administering a drug by an eluting balloon. This approach would take advantage prolonging the drug residence time in the injured artery by administering drug-eluting NPs. Indeed, the efficacy could rely on both the drug released into cells after NP uptake and the drug passively released in the extracellular matrix. Furthermore, SMCs represent a promising pharmacological target because their migration should be preferentially blocked to avoid the neointima formation; at the same time, endothelial cells should be preserved because their migration favors the safety of the vessel. This work presents a proof-of-concept of FLV-NP which shows the potentiality to inhibit the SMC migration, without altering the FLV effect on proliferation and migration of endothelial cells at the same NP concentration. Data on in vitro experiments showed that the endothelial cell vitality was significantly reduced when treated with a 1 μM FLV solution, while that of SMCs was unaffected. Conversely, when the two cell lines were treated with FLV-NP, SMCs appeared more sensible to FLV treatment than endothelial cells because SMC proliferation was significantly inhibited at 4 μM, while the toxic effect in endothelial cells was observed only at a higher concentration (10 μM).

In conclusion, the improved trophism on NPs toward SMCs, combined with the excellent biocompatibility and low modification of microenvironment pH upon polymer degradation, makes *g-*CA-PLGA a good candidate for the design of DEBs.
